# Prevalence and predictors of depressive symptoms among HIV-positive men who inject drugs in Vietnam

**DOI:** 10.1371/journal.pone.0191548

**Published:** 2018-01-24

**Authors:** Sara N. Levintow, Brian W. Pence, Tran Viet Ha, Nguyen Le Minh, Teerada Sripaipan, Carl A. Latkin, Pham The Vu, Vu Minh Quan, Constantine Frangakis, Vivian F. Go

**Affiliations:** 1 University of North Carolina, Gillings School of Global Public Health, Department of Epidemiology, Chapel Hill, NC, United States of America; 2 University of North Carolina, Gillings School of Global Public Health, Department of Health Behavior, Chapel Hill, NC, United States of America; 3 Thai Nguyen Center for Preventive Medicine, Thai Nguyen, Vietnam; 4 Johns Hopkins Bloomberg School of Public Health, Department of Health, Behavior and Society, Baltimore, MD, United States of America; 5 Johns Hopkins Bloomberg School of Public Health, Department of Epidemiology, Baltimore, MD, United States of America; 6 Johns Hopkins Bloomberg School of Public Health, Department of Biostatistics, Baltimore, MD, United States of America; Brown University, UNITED STATES

## Abstract

**Background:**

HIV infection is common among people who inject drugs (PWID), and HIV-positive PWID may be particularly vulnerable to depression. This study measured the prevalence of depressive symptoms and the factors associated with severe symptoms among 455 HIV-positive PWID in Thai Nguyen, Vietnam.

**Methods:**

We used cross-sectional data from PWID in a randomized controlled trial of an intervention to reduce high-risk injecting and sexual behaviors in Thai Nguyen from 2009–2013. Depressive symptoms were measured with the Center for Epidemiologic Studies Depression Scale (CES-D). We used logistic regression to assess demographic, clinical, and psychosocial predictors of severe depressive symptoms (CES-D≥23) with prevalence odds ratios (POR) and 95% confidence intervals (CI).

**Results:**

The prevalence of severe depressive symptoms (CES-D≥23) was 44%. 25% of participants had mild to moderate depressive symptoms (16≤CES-D<23), and 31% experienced no depressive symptoms (CES-D<16). Not being married, self-rated poor health, greater frequency of injection drug use, history of overdose, no alcohol use, and daily cigarette smoking were positively associated with severe depressive symptoms in unadjusted models and remained predictive in a multivariable model. The strongest predictors of depressive symptoms were self-reported poor health (POR = 2.94, 95% CI: 1.82, 4.76), no current alcohol use (POR = 2.35, 95% CI: 1.47, 3.77), and not currently married or cohabitating (POR = 2.21, 95% CI = 1.40, 3.47).

**Conclusion:**

Severe depressive symptoms were common among HIV-positive PWID in Thai Nguyen and were strongly associated with demographic, clinical, and psychosocial factors. Interventions that promote social support from family and reduce drug dependence may particularly benefit PWID experiencing severe depressive symptoms. Greater recognition and treatment of depressive symptoms has the potential to enhance quality of life and improve HIV clinical outcomes for PWID.

## Introduction

Injection drug use carries a high risk of HIV transmission and is the driver of the HIV epidemic in parts of Asia and Eastern Europe [1]. Out of an estimated 12.2 million people who inject drugs (PWID) worldwide, 1.6 million (13.5%) are living with HIV [2], and in Vietnam, up to 40% of PWID may be HIV-positive, varying by province [3,4]. Thai Nguyen is a northeastern province close to the border with China where urbanization and easily accessible opium and heroin have resulted in increasing injection drug use since the 1990s. Among approximately 6,000 PWID in the province, an estimated 34% are living with HIV [5].

Depression is common among people living with HIV [6,7], and HIV-positive PWID may be particularly vulnerable to depression [8–11]. The physical and psychological consequences of living with both HIV and drug addiction can lead to and exacerbate depressive symptoms. Depression, in turn, has a negative impact on HIV clinical progression [12]. Compared to patients without depression, depressed HIV patients have worsened immune function [13], decreased adherence to antiretroviral therapy (ART) [14], slower viral suppression [15], faster progression to AIDS [16], and higher risk of mortality [17,18]. The co-occurrence of both depression and injection drug use among people living with HIV results in the poorest outcomes [10,19].

Early identification of depressive symptoms among HIV-positive PWID is critical to improving health outcomes in this vulnerable population. Addressing depressive symptoms as part of HIV care could enhance quality of life and lessen the impact of depression on HIV clinical progression [20]. Although studies on depression and HIV are numerous, research is limited on the factors that contribute to depression among HIV-positive PWID. Previous studies on depression among HIV-positive PWID have occurred in the US [21–23] and Switzerland [11] where HIV is not concentrated among PWID and unmet needs for mental health services are not as great. Prior studies in more comparable settings in Indonesia and China focused on PWID not known to be HIV-positive [8] or enrolled participants who were engaged in HIV care and no longer active drug users [9]. To date, studies in Vietnam have assessed the prevalence of depressive symptoms in HIV-positive populations, with estimates ranging from 19% to 40% [24–28]. However, none of these studies has focused on HIV-positive PWID, and the prevalence in this high risk group remains unknown.

While understanding the prevalence of depression in this population is important, addressing the burden of depression requires efficient targeting of resources. Mental illness is beyond the traditional scope of HIV physicians in Vietnam, and depressive symptoms are often overlooked at outpatient HIV clinics [25,29,30]. Training clinic staff to identify depressive symptoms is challenging in the resource-limited settings of Vietnam [25]. Use of depression screening tools, such as the Center for Epidemiologic Studies Depression Scale (CES-D), provide a cost-effective method for recognizing depressive symptoms. Clinic staff without specialized psychiatric training can identify patients for referral to services under the Vietnam national mental health program. Furthermore, determining the characteristics of patients who experience severe depressive symptoms will inform the development and targeting of mental health interventions. A greater understanding of these factors will help to focus limited resources on those most in need of mental health care.

To address our limited understanding of depression in this high risk population, this study measures the prevalence of depressive symptoms among HIV-positive PWID in Thai Nguyen, Vietnam. We further sought to identify demographic, clinical, and psychosocial factors that were associated with severe depressive symptoms in this population. Recognizing depressive symptoms is critical to improving HIV outcomes among PWID and addressing the burden of depression in the resource-limited settings of the HIV epidemic.

## Methods

### Study population

Our analysis used cross-sectional baseline data from a randomized controlled trial of an intervention to reduce high-risk injecting and sexual behaviors among HIV-positive PWID in Thai Nguyen from 2009–2013. Details of the trial and primary outcomes have been reported elsewhere [31]. Briefly, Thai Nguyen is a northeastern province in Vietnam with increases in injection drug use since the 1990s, and the trial enrolled participants in the 32 sub-districts of Thai Nguyen with the highest number of PWID. Participant recruitment was conducted using snowball sampling, in which recruiters who were former and current drug users approached members of drug networks in private places to discuss study enrollment. The trial enrolled 455 participants who met the following criteria: 1) HIV-positive diagnosis confirmed through study testing, 2) male (given that 97% of PWID in Thai Nguyen are male), 3) at least 18 years old, 4) had sex in the past 6 months, 5) injected drugs in the previous six months, and 6) planned to live in Thai Nguyen for the next 24 months. The trial and this analysis were approved by the ethical review committees at the Thai Nguyen Center for Preventive Medicine, the Johns Hopkins Bloomberg School of Public Health, and the University of North Carolina at Chapel Hill School of Public Health. Written informed consent was obtained from all participants.

### Conceptual model and hypotheses

This study assessed the prevalence of depressive symptoms and identified factors associated with depression at the baseline visit for the trial. Our conceptual framework of depression among HIV-positive PWID was based on the biopsychosocial model and considered demographic, clinical, and psychosocial factors as determinants of depression [32]. These factors have been well studied in the general population and in some HIV-positive populations, with limited research among PWID or in Vietnam [33,34]. This study evaluated the relationship between the demographic, clinical, and psychosocial variables measured in the trial and the likelihood of participants reporting depressive symptoms.

Demographic characteristics shape living and working conditions of daily life and have been found to affect the prevalence of depression in HIV-positive populations [8,9,11,35]. In this study, younger age, lower education, unemployment, and not being married were hypothesized to be associated with a higher likelihood of depressive symptoms among PWID. Clinical factors capture aspects of physical health, perceived by the individual or measured directly, that can lead to and be affected by depression [6,23,34,36,37]. Among HIV-positive PWID, poorer general health, lower CD4 cell count, awareness of HIV-positive status, and not taking ART were expected to be associated with depressive symptoms. Finally, psychosocial factors play a critical role in the experience of depressive symptoms, with prior research linking social support, stigma, drug use, and quality of life to depression in HIV-positive [21,38,39] and uninfected PWID [8,22]. A lower level of social support, greater stigma, and higher frequency of substance use were hypothesized to be associated with depressive symptoms.

### Measures

At the baseline visit for the trial, participants received HIV testing and were administered a questionnaire prior to receipt of HIV results. The questionnaire collected information on demographic, clinical, and psychosocial factors. As part of the questionnaire, the CES-D was administered, which is a 20-item screening tool and widely used measure of depressive symptoms experienced over the past week [40]. Previous studies have demonstrated that the CES-D is a valid and reliable measure of depressive symptoms in Vietnamese populations [25,41–43]. Although the CES-D is not diagnostic of clinical depression, a score of 16 or greater has been found to indicate probable depressive symptoms among the general population, and higher cut-points have been used among patients with comorbid chronic illness, such as HIV [25]. Given our focus on HIV-positive PWID, we assessed the typical cut-point of 16 in addition to a more conservative score of 23 to indicate severe depressive symptoms.

#### Demographic variables

The questionnaire collected information on participants’ age, education, employment, and marital status.

#### Clinical variables

In the questionnaire, participants rated their general health on a five-point scale from “Excellent” to “Poor.” Participants were asked about prior HIV testing, previous positive results, and ART use in the prior six months. They also provided a blood specimen to assess CD4 cell count.

#### Psychosocial variables

The questionnaire collected information on participants’ perceptions of social support and stigma and their behaviors related to substance use.

Social support was assessed using a modified version of the Medical Outcomes Study (MOS) social support scale that was developed by Sherbourne and Stewart and found to be reliable and stable over time [44]. The scale contained 20 items, asking for the number of close friends and relatives and the types of social support available to the respondent. The four MOS subscales were found to be highly correlated (ρ = 0.43 to 0.80) and had similar associations with depressive symptoms. For this analysis, instead of the individual subscale scores, an overall social support score was calculated for each participant.

Participants were asked about perceived, internalized, and experienced stigma related to both HIV and injection drug use. A set of four stigma scales with a total of 40 items had been developed for the trial, and details have been reported elsewhere [45]. In this analysis, an overall HIV stigma score and an overall drug use stigma score were calculated for each participant.

Participants were asked about the type of drug most frequently injected, the number of days during the past three months they injected that drug, and if they had ever overdosed. Participants completed the Alcohol Use Disorders Identification Test (AUDIT), a screening tool developed by the World Health Organization that assesses recent alcohol use, dependence symptoms, and alcohol-related problems (score of 8 or more) [46]. This analysis used the AUDIT score to classify participants as reporting no current alcohol use (AUDIT score = 0), reporting some alcohol use that was not harmful (AUDIT score = 1–7), and reporting harmful alcohol use (AUDIT score ≥ 8). Cigarette smoking was assessed by asking participants how many days a week they smoked cigarettes over the last three months.

### Statistical analysis

The prevalence of depressive symptoms was assessed using the CES-D scores. Based on the CES-D cutpoints evaluated in prior research [25,40], we classified scores of 23 or greater as indicating severe depressive symptoms, scores of 16–22 as mild to moderate depressive symptoms, and scores less than 16 to indicate no symptoms. Demographic, clinical, and psychosocial variables were summarized using means with standard deviations for continuous variables and using frequencies and proportions for categorical variables.

We developed a multivariable model to predict the probability of severe depressive symptoms (CES-D score ≥ 23) among PWID. Logistic regression models estimated the prevalence odds ratio (POR) and 95% confidence interval for the crude association between severe depressive symptoms and each variable. All variables with a p-value ≤0.25 were included in the multivariable model, and correlations between covariates were assessed, with no collinearity problems identified. For continuous variables, functional form was assessed based on model fit and likelihood ratio tests. Linear specifications were selected for age, social support score, and stigma scores, and we used CD4 categories based on the CDC classification system of HIV clinical stages. The multivariable model examined the independent effect of each variable on depressive symptoms after adjustment for all other variables. We assessed both the magnitude of the association and the statistical significance of variables in the multivariable model. We considered variables to have a meaningful association with depressive symptoms if the POR was ≤0.7 or ≥1.5 with a p-value <0.10. While we identified all variables fulfilling these criteria to suggest important associations, we also noted those variables that had 95% CI that excluded the null (p-value <0.5).

All analyses were conducted using SAS Version 9.4 (SAS Institute Inc., Cary, NC) and R Version 3.3.1 (R Foundation for Statistical Computing, Vienna, Austria).

## Results

Baseline characteristics of the 455 HIV-positive PWID enrolled in the trial are shown in Table 1. The average age of participants was 35 years, half were married or cohabitating (47%), and most had at least a secondary school education (89%) and were employed full-time or part-time (87%). Most participants did not know their HIV-positive status (72%), and given that the majority were newly diagnosed, few were taking ART at baseline (13%). The majority had a CD4 cell count between 200 and 500 cells/μL (48%) or less than 200 cells/μL (41%). Most rated their general health as fair (66%) or poor (30%). All participants reported injecting heroin in the past three months, half reported injecting heroin daily (51%), and most did not report a history of overdose (82%). The majority reported current alcohol use (67%) and daily cigarette smoking (86%). Based on the trial inclusion criteria, all were male and HIV-positive.

**Table 1:** Characteristics of 455 HIV-positive PWID in Thai Nguyen, Vietnam 2009–2013.

**Demographic Factors**	**Mean (SD) or N (%)**
Age in years (range 19–60)	35 (6)
Education	
Less than secondary school	50 (11)
Secondary school, less than high school	252 (55)
High school or greater	153 (34)
Employment status	
Currently unemployed or unable to work	58 (13)
Working full-time or part-time	397 (87)
Marital status	
Single	175 (38)
Widowed, divorced, or separated	65 (14)
Married or cohabitating	215 (47)
**Clinical Factors**	**Mean (SD) or N (%)**
Self-rated health	
Excellent	0 (0)
Very Good	2 (1)
Good	16 (4)
Fair	301 (66)
Poor	136 (30)
HIV+ status knowledge/ART^a^	
Don’t know status, not on ART	323 (72)
Know status, not on ART	68 (15)
Know status and on ART	58 (13)
CD4 cell count (cells/μL)^a^	
≥500	51 (11)
200–499	214 (48)
<200	181 (41)
**Psychosocial Factors**	**Mean (SD) or N (%)**
Injection drug use^a^	
Inject heroin daily	224 (51)
Inject heroin less than daily	219 (49)
History of overdose	
Never overdosed	371 (82)
Overdosed in lifetime, not in past year	61 (13)
Overdosed in past year	23 (5)
Alcohol use	
No alcohol use (AUDIT = 0)	148 (33)
Some alcohol use, but not harmful (AUDIT = 1–7)	216 (47)
Harmful alcohol use (AUDIT ≥ 8)	91 (20)
Cigarette smoking	
Smoke cigarettes 7 days/week	391 (86)
Smoke cigarettes less than 7 days/week	64 (14)
HIV stigma score (range 14–44)^b^	30 (4)
Injection drug use stigma score (range 9–28)^b^	19 (3)
Social support score (range 0–400)^c^	275 (83)

^a^ CD4 cell count was missing for 9 participants (2%), ART status was missing for 6 participants (1%), and frequency of injection drug use was missing for 12 participants (3%).

^b^ Stigma scores were calculated with stigma scales on perceived, internalized, and experienced stigma previously developed for the study population.

^c^ Social support was calculated using a modified version of the MOS social support scale. For all variables, percentages may not sum to 100 due to rounding.

**Fig 1:**
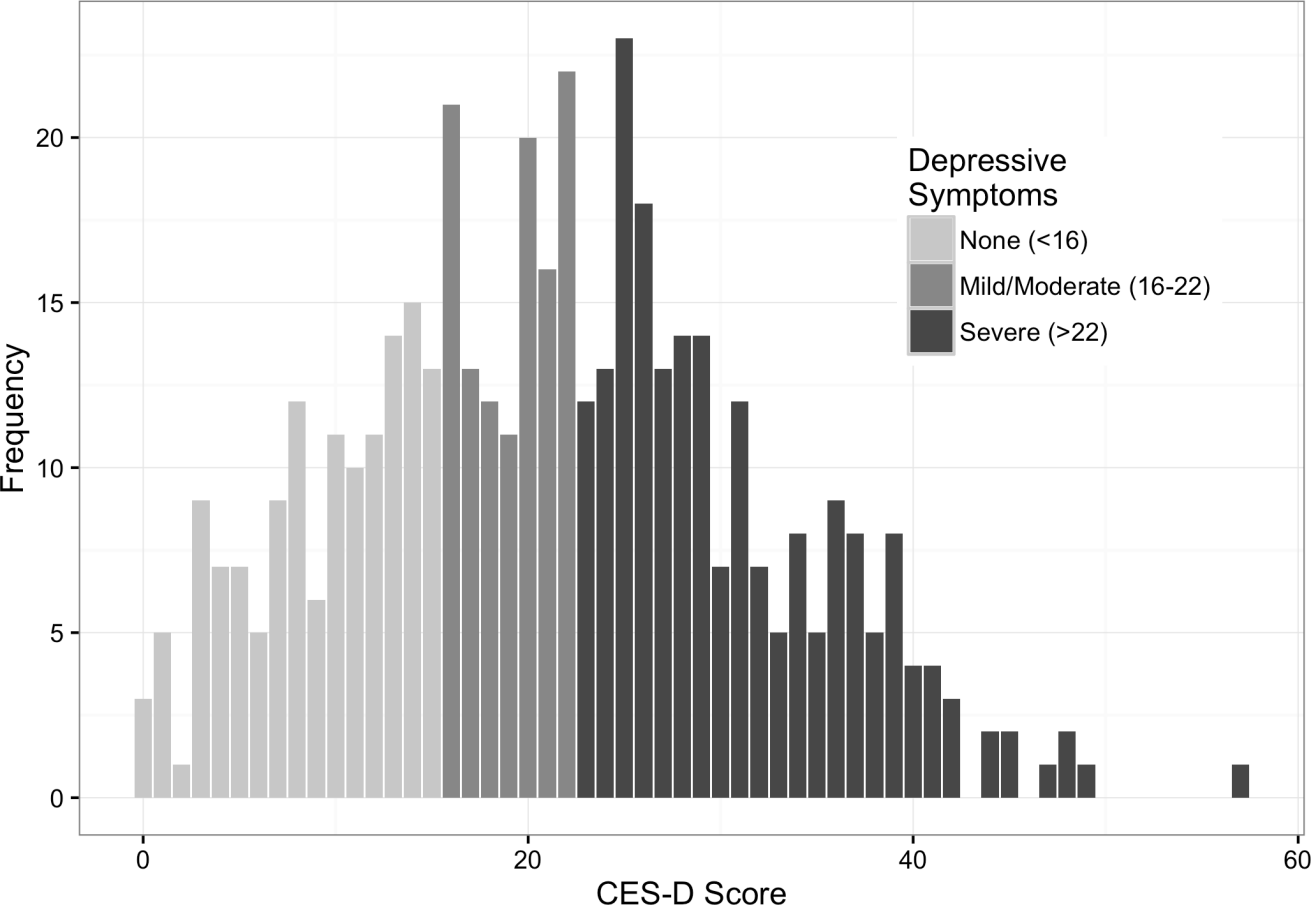
Distribution of CES-D scores among 455 HIV-positive PWID in Thai Nguyen, Vietnam 2009–2013.

Fig 1 shows the distribution of CES-D scores among participants in the trial. CES-D scores ranged from 0 to 57 (out of a maximum score of 60), and the average score was 21.3 (SD = 10.5). Among the 455 participants, the prevalence of severe depressive symptoms was 44%, with 201 participants scoring a 23 or greater on the CES-D. 115 participants (25%) reported mild to moderate depressive symptoms (16≤CES-D<23), and 139 participants (30%) experienced no depressive symptoms (CES-D<16).

The associations between severe depressive symptoms (CES-D≥23) and demographic, clinical, and psychosocial factors are shown in Table 2. In unadjusted models, all variables except for CD4 cell count and age were associated with depressive symptoms and were included in the multivariable model (p<0.25). In the multivariable analysis, marital status, self-rated health, injection drug use, history of overdose, alcohol use, and cigarette smoking remained independently associated with depressive symptoms (p < 0.10 and POR ≤0.7 or ≥1.5). The strongest predictors of depressive symptoms were self-reported poor health (POR = 3.32, 95% CI 2.08, 5.30), no current alcohol use (POR = 2.58, 95% CI: 1.63, 4.08), and not currently married or cohabitating (POR = 2.36, 95% CI = 1.52, 3.64).

**Table 2:** Associations between depressive symptoms and demographic, clinical, and psychosocial factors at study baseline among HIV-positive PWID in Vietnam.

**Demographic Factors**	**Unadjusted** **POR (95% CI)**	**Full Model** **POR (95% CI)**
Age		
10-year change in age	0.95 (0.71, 1.27)	—
Education		
Secondary school or greater	1.00	1.00
Less than secondary school	1.87 (1.03, 3.39)^a^	1.74 (0.85, 3.56)
Employment status		
Working full-time or part-time	1.00	1.00
Unemployed or unable to work	1.42 (0.82, 2.46)^a^	0.98 (0.52, 1.88)
Marital status		
Married or cohabitating	1.00	1.00
Single	2.16 (1.43, 3.25)^a^	2.01 (1.23, 3.28)^c^
Widowed, divorced, or separated	2.41 (1.37, 4.25)^a^	2.25 (1.18, 4.27)^c^
**Clinical Factors**	**Unadjusted** **POR (95% CI)**	**Full Model** **POR (95% CI)**
Self-rated health		
Very Good, Good, Fair	1.00	1.00
Poor	3.84 (2.51, 5.88)^a^	2.94 (1.82, 4.76)^c^
HIV+ status knowledge/ART		
Don’t know status, not on ART	1.00	1.00
Know status, not on ART	2.11 (1.24, 3.61)^a^	1.38 (0.73, 2.58)
Know status and on ART	0.85 (0.48, 1.51)	1.03 (0.52, 2.03)
CD4 cell count (cells/μL)		
≥500	1.00	—
200–499	1.05 (0.57, 1.95)	—
<200	1.04 (0.56, 1.95)	—
**Psychosocial Factors**	**Unadjusted** **POR (95% CI)**	**Full Model** **POR (95% CI)**
Injection drug use		
Inject heroin less than daily	1.00	1.00
Inject heroin daily	2.09 (1.42, 3.05)^a^	1.54 (0.98, 2.42)^b^
History of overdose		
Never overdosed	1.00	1.00
Ever overdosed, not past year	1.70 (0.99, 2.93)^a^	1.72 (0.91, 3.27)^b^
Overdosed in past year	3.29 (1.32, 8.20)^a^	2.95 (1.00, 8.76)^b^
Alcohol use		
No alcohol use	1.00	1.00
Some alcohol use, not harmful	0.33 (0.21, 0.51)^a^	0.42 (0.25, 0.69)^c^
Harmful alcohol use	0.31 (0.18, 0.53)^a^	0.44 (0.23, 0.84)^c^
Cigarette smoking		
Smoke cigarettes less than daily	1.00	1.00
Smoke cigarettes daily	1.90 (1.08, 3.34)^a^	1.75 (0.91, 3.37)^b^
HIV stigma		
1 SD change in score	1.14 (0.94, 1.37)^a^	1.10 (0.88, 1.39)
Injection drug use stigma		
1 SD change in score	1.31 (1.08, 1.58)^a^	1.15 (0.92, 1.44)
Social support		
1 SD change in score	0.66 (0.54, 0.80)^a^	0.82 (0.65, 1.02)^b^

^a^ p ≤ 0.25

^b^ 0.05 ≤ p ≤ 0.10

^c^ p < 0.05

## Discussion

Our study found that high levels of depressive symptoms were common among HIV-positive PWID, with 44% reporting severe symptoms from the previous week. This estimate is consistent with previous reports on depression prevalence among people living with HIV of 40–42% worldwide [34]. Prior studies measuring prevalence in Vietnam found a range from 19% to 40% of people living with HIV reported current depressive symptoms [24–28,47]. The elevated prevalence of severe depressive symptoms in our study among HIV-positive PWID, compared to prior work that did not focus on PWID, supports our hypothesis that PWID are particularly vulnerable to depression. They experience a higher burden of depression compared to other key populations in Vietnam, such as men who have sex with men [48]. Notably, our study used a conservative CES-D score of 23 or greater to indicate severe depressive symptoms. An even higher percentage (69%) of participants would be classified as experiencing depressive symptoms based on the cut-point of 16 typically used in the general population. In addition, most participants (72%) were unaware of their HIV-positive status when they responded to the CES-D, and depressive symptoms may worsen after learning their diagnosis.

Nearly all demographic, clinical, and psychosocial factors were associated with severe depressive symptoms in unadjusted models. Consistent with prior research, marital status, self-rated health, injection drug use, and cigarette smoking remained predictive in multivariable models. Knowledge of these factors associated with depression can inform the development and targeting of mental health interventions for PWID. For example, interventions that promote emotional and tangible support from family members may be optimal. Reducing drug dependency and addressing basic medical needs are also critical components for future work, potentially through linkage to methadone maintenance therapy or HIV outpatient clinics.

An unexpected finding was the inverse relationship between alcohol use and depressive symptoms. Alcohol use, regardless of frequency, appeared protective against depressive symptoms, and no alcohol use was a strong predictor of depressive symptoms among our study’s participants. Our qualitative research in this population indicates that alcohol use may be a proxy for social support and participation in family and community gatherings, with no alcohol use corresponding to social isolation and loneliness [49]. We found support for a positive relationship between alcohol use and social support in our study. The POR for alcohol use associated with a 1 SD increase in social support score was 1.36 (95% CI: 1.12, 1.66), and the correlation between participants’ AUDIT score and social support score was ρ = 0.17 (p < 0.01). Another explanation is that participants with higher drug use and depressive symptoms purchase heroin frequently and lack money to spend on alcohol. We found that the POR for alcohol use associated with daily injection drug use was 0.47 (95% CI: 0.31, 0.70), indicating that frequent drug use was indeed associated with no alcohol consumption. This inverse relationship between injection drug use and alcohol consumption is also consistent with prior research [50,51].

This is the first study of depression among HIV-positive PWID in Vietnam, but an important limitation is that participants were not randomly sampled and may differ from other HIV-positive PWID in Vietnam not recruited for this study. Due to snowball sampling, our study conclusions are specific to this sample and should not be inferred as representative of the population. It is also important to note that our findings may not be applicable to other groups, particularly women, or settings where the HIV epidemic is not concentrated among PWID. However, male PWID in Vietnam are a critical population to study. They are the driver of the HIV epidemic in Vietnam, and one-third of PWID in Thai Nguyen are HIV-positive. Our findings may also be relevant to other Asian and Eastern European countries where the HIV epidemic is concentrated among similar groups of men who inject drugs.

Another limitation of our study is that the CES-D corresponds to probable depression, not a clinical diagnosis. It does not include items on suicidal ideation, which is an important domain for assessing clinical depression. The CES-D may be affected by social desirability bias or recall bias, such that participants’ scores are not a true indication of their underlying depression. However, validation studies have found that the CES-D has high reliability and validity when compared with clinical assessments made by psychiatrists [25]. In addition to its strong psychometric properties, clinic staff with no specialized training can use the CES-D to identify patients with probable depression for further evaluation and referral to mental health services.

Our findings demonstrate that severe depressive symptoms are common among HIV-positive PWID in Thai Nguyen and can be predicted by a variety of demographic, clinical, and psychosocial factors. Given this high prevalence, mental health interventions and widespread availability of depression treatment are urgently needed in Vietnam. Interventions focusing on family social support, drug dependency, and linkage to care may be particularly beneficial for PWID. Ultimately, addressing depression as part of HIV care could improve health outcomes for both of these comorbid and debilitating conditions. Further research is important to identify effective approaches to reducing depressive symptoms and subsequently improving HIV outcomes.

## Supporting information

S1 Dataset Supporting information Dataset S1. (CSV)

## References

[1] Dutta A, Wirtz A, Stanciole A, Oelrichs R, Semini I, Baral S, et al The global HIV epidemics among people who inject drugs. Washington DC: World Bank; 2013.

[2] United Nations Office on Drugs and Crime. World Drug Report New York; 2015.

[3] Mathers BM, Degenhardt L, Phillips B, Wiessing L, Hickman M, Strathdee SA, et al Global epidemiology of injecting drug use and HIV among people who inject drugs: a systematic review. Lancet. 2008; 372: p. 1733–45. doi: 10.1016/S0140-6736(08)61311-2 18817968 10.1016/S0140-6736(08)61311-2

[4] Ministry of Health of Vietnam. Results from the HIV/STI integrated biological and behavioral surveillance (IBBS) in Vietnam, Round II, 2009 Vietnam: Ministry of Health; 2011.

[5] Socialist Republic of Viet Nam. Vietnam AIDS response progress report 2014, following up the 2011 political declaration on HIV/AIDS, reporting period: January 2013-December 2013 Hanoi, Vietnam: National Committee for AIDS, Drugs, and Prostitution Prevention and Control; 2014.

[6] Treisman G, Angelino A. Interrelation between psychiatric disorders and the prevention and treatment of HIV infection. Clin Infect Dis. 2007; 45: p. S313–7. doi: 10.1086/522556 18190305 10.1086/522556

[7] Bing EG, Burnam MA, Longshore D, Fleishman JA, Sherbourne CD, London AS, et al Psychiatric disorders and drug use among human immunodeficiency virus-infected adults in the United States. Arch Gen Psychiatry. 2001; 58: p. 721–8. 11483137 10.1001/archpsyc.58.8.721

[8] Li J, Gu J, Lau JTF, Chen H, Mo PKH, Tang M. Prevalence of depressive symptoms and associated factors among people who inject drugs in China. Drug Alcohol Depend. 2015; 151: p. 228–35. doi: 10.1016/j.drugalcdep.2015.03.028 25920800 10.1016/j.drugalcdep.2015.03.028

[9] Li Y, Hershow R, Praptoraharjo I, Setiawan M, Levy J. Factors associated with symptoms of depression among injection drug users receiving antiretroviral treatment in Indonesia. J AIDS Clin Res. 2014; 5: 303–. doi: 10.4172/2155-6113.1000303 25328813 10.4172/2155-6113.1000303 PMC4198157

[10] Bouhnik AD, Preau M, Vincent E, Carrieri MP, Gallais H, Lepeu G, et al Depression and clinical progression in HIV-infected drug users treated with highly active antiretroviral therapy. Antivir Ther. 2005; 10: p. 53–61. 15751763

[11] Anagnostopoulos A, Ledergerber B, Jaccard R, Shaw SA, Stoeckle M, Bernasconi E, et al Frequency of and risk factors for depression among participants in the swiss HIV Cohort Study (SHCS). PLoS One. 2015; 10: p. 1–17. 10.1371/journal.pone.0140943 PMC4619594 26492488

[12] Leserman J. Role of depression, stress, and trauma in HIV disease progression. Psychosom Med. 2008; 70: p. 539–45. doi: 10.1097/PSY.0b013e3181777a5f 18519880 10.1097/PSY.0b013e3181777a5f

[13] Ironson G, O’Cleirigh C, Fletcher MA, Laurenceau JP, Balbin E, Klimas N, et al Psychosocial factors predict CD4 and viral load change in men and women with human immunodeficiency virus in the era of highly active antiretroviral treatment. Psychosom Med. 2005; 67: p. 1013–21. doi: 10.1097/01.psy.0000188569.58998.c8 16314608 10.1097/01.psy.0000188569.58998.c8 PMC2614887

[14] Gordillo V, del Amo J, Soriano V, González-Lahoz J. Sociodemographic and psychological variables influencing adherence to antiretroviral therapy. AIDS. 1999; 13: p. 1763–9. 10509579 10.1097/00002030-199909100-00021

[15] Pence BW, Miller WC, Gaynes BN, Eron JJ. Psychiatric illness and virologic response in patients initiating highly active antiretroviral therapy. J Acquir Immune Defic Syndr. 2007; 44: p. 159–66. doi: 10.1097/QAI.0b013e31802c2f51 17146374 10.1097/QAI.0b013e31802c2f51

[16] Cook JA, Grey D, Burke J, Cohen MH, Gurtman AC, Richardson JL, et al Depressive symptoms and AIDS-related mortality among a multisite cohort of HIV-positive women. Am J Public Health. 2004; 94: p. 1133–40. 15226133 10.2105/ajph.94.7.1133 PMC1448411

[17] Ickovics JR, Hamburger ME, Vlahov D, Schoenbaum EE, Schuman P, Boland RJ, et al Mortality, CD4 cell count decline, and depressive symptoms among HIV-seropositive women: longitudinal analysis from the HIV Epidemiology Research Study. JAMA. 2001; 285: p. 1466–74. 11255423 10.1001/jama.285.11.1466

[18] Leserman J, Pence BW, Whetten K, Mugavero MJ, Thielman NM, Swartz MS, et al Relation of lifetime trauma and depressive symptoms to mortality in HIV. Am J Psychiatry. 2007; 164: p. 1707–13. doi: 10.1176/appi.ajp.2007.06111775 17974936 10.1176/appi.ajp.2007.06111775

[19] Milloy MJ, Marshall BDL, Kerr T, Buxton J, Rhodes T, Montaner J, et al Social and structural factors associated with HIV disease progression among illicit drug users: a systematic review. AIDS. 2012; 26: p. 1049–63. doi: 10.1097/QAD.0b013e32835221cc 22333747 10.1097/QAD.0b013e32835221cc PMC3955099

[20] Sherr L, Clucas C, Harding R, Sibley E, Catalan J. HIV and depression: a systematic review of interventions. Psychol Heal Med. 2011; 16: p. 493–527. 10.1080/13548506.2011.579990 21809936

[21] Valverde EE, Purcell DW, Waldrop-Valverde D, Farrell N, Malow R, Knowlton AR, et al Correlates of depression among HIV-positive women and men who inject drugs. J Acquir Immune Defic Syndr. 2007; 46: p. S96–100. doi: 10.1097/QAI.0b013e318157683b 18089990 10.1097/QAI.0b013e318157683b

[22] Knowlton AR, Latkin CA, Schroeder JR, Hoover DR, Ensminger M, Celentano DD. Longitudinal predictors of depressive symptoms among low income injection drug users. AIDS Care. 2001; 13: p. 549–59. doi: 10.1080/09540120120063197 11571003 10.1080/09540120120063197

[23] Springer SA, Chen S, Altice F. Depression and symptomatic response among HIV-infected drug users enrolled in a randomized controlled trial of directly administered antiretroviral therapy. AIDS Care. 2009; 21: p. 976–983. doi: 10.1080/09540120802657555 20024753 10.1080/09540120802657555 PMC2797133

[24] Esposito CA, Steel Z, Gioi TM, Huyen TTN, Tarantola D. The prevalence of depression among men living with HIV infection in Vietnam. Am J Public Health. 2009; 99: p. S439–44. doi: 10.2105/AJPH.2008.155168 19797756 10.2105/AJPH.2008.155168 PMC4504376

[25] Thai TT, Jones MK, Harris LM, Heard RC. Screening value of the Center for Epidemiologic Studies—Depression Scale among people living with HIV/AIDS in Ho Chi Minh City, Vietnam: a validation study. BMC Psychiatry. 2016;16(1):145. 27178070 10.1186/s12888-016-0860-3 PMC4868017

[26] Green K, Tuan T, Hoang TV, Thi Trang NN, Thanh Ha NT, Hung ND. Integrating palliative care into HIV outpatient clinical settings: Preliminary findings from an intervention study in vietnam. J Pain Symptom Manage. 2010; 40: p. 31–4. doi: 10.1016/j.jpainsymman.2010.04.006 20619211 10.1016/j.jpainsymman.2010.04.006

[27] Huynh V-AN, To KG, Do D Van, To QG, Nguyen MTH. Changes in depressive symptoms and correlates in HIV+ people at An Hoa Clinic in Ho Chi Minh City, Vietnam. BMC Psychiatry. 2017;17(1): p. 35 doi: 10.1186/s12888-016-1170-5 28109260 10.1186/s12888-016-1170-5 PMC5251339

[28] Thai TT, Jones MK, Harris LM, Heard RC. The association between symptoms of mental disorders and health risk behaviours in Vietnamese HIV positive outpatients: a cross-sectional study. BMC Public Health. 2017; 17(1): p. 250 doi: 10.1186/s12889-017-4162-6 28288615 10.1186/s12889-017-4162-6 PMC5348739

[29] Ministry of Health of Vietnam. WHO-AIMS report on mental health system in Vietnam World Health Organization; 2006.

[30] Vuong DA, Van Ginneken E, Morris J, Ha ST, Busse R. Mental health in Vietnam: Burden of disease and availability of services. Asian J Psychiatr. 2011; 4: p. 65–70. doi: 10.1016/j.ajp.2011.01.005 23050918 10.1016/j.ajp.2011.01.005

[31] Go VF, Frangakis C, Minh N Le, Latkin C, Ha TV, Mo TT, et al Efficacy of a multi-level intervention to reduce injecting and sexual risk behaviors among HIV-infected people who inject drugs in Vietnam: a four-arm randomized controlled trial. PLoS One. 2015; 10: p. e0125909 doi: 10.1371/journal.pone.0125909 26011427 10.1371/journal.pone.0125909 PMC4444299

[32] Engel GL. The clinical application of the biopsychosocial model. Am J Psychiatry. 1980; 137: p. 535–44. doi: 10.1176/ajp.137.5.535 7369396 10.1176/ajp.137.5.535

[33] Kessler RC, Bromet EJ. The epidemiology of depression across cultures. Annu Rev Public Health. 2013; 34: p. 119–38. doi: 10.1146/annurev-publhealth-031912-114409 23514317 10.1146/annurev-publhealth-031912-114409 PMC4100461

[34] Nanni MG, Caruso R, Mitchell AJ, Meggiolaro E, Grassi L. Depression in HIV infected patients: a review. Curr Psychiatry Rep. 2015; 17: p. 530 doi: 10.1007/s11920-014-0530-4 25413636 10.1007/s11920-014-0530-4

[35] Nomoto SH, Longhi RM, de Barros BP, Croda J, Ziff EB, Castelon KE. Socioeconomic disadvantage increasing risk for depression among recently diagnosed HIV patients in an urban area in Brazil: cross-sectional study. AIDS Care. 2015; 27: p. 979–85. doi: 10.1080/09540121.2015.1017442 25741909 10.1080/09540121.2015.1017442

[36] Evans DL, Leserman J, Pedersen CA, Golden RN, Lewis MH, Folds JA, et al Immune correlates of stress and depression. Psychopharmacol Bull. 1989; 25: p. 319–24. 2697005

[37] Herbert TB, Cohen S. Depression and immunity: a meta-analytic review. Psychol Bull. 1993; 113: p. 472–86. 8316610 10.1037/0033-2909.113.3.472

[38] Mizuno Y, Purcell DW, Dawson-Rose C, Parsons JT. Correlates of depressive symptoms among HIV-positive injection drug users: the role of social support. AIDS Care. 2003; 15: p. 689–98. doi: 10.1080/09540120310001595177 12959820 10.1080/09540120310001595177

[39] Semple SJ, Strathdee SA, Zians J, Patterson TL. Factors associated with experiences of stigma in a sample of HIV-positive, methamphetamine-using men who have sex with men. Drug Alcohol Depend. 2012; 125: p. 154–9. doi: 10.1016/j.drugalcdep.2012.04.007 22572209 10.1016/j.drugalcdep.2012.04.007 PMC3419298

[40] Radloff LS. A self-report depression scale for research in the general population. Appl Psychol Meas. 1977; 1: p. 385–401.

[41] Franks F, Faux SA. Depression, stress, mastery, and social resources in four ethnocultural women’s groups. Res Nurs Health. 1990; 13: p. 283–92. 2236651 10.1002/nur.4770130504

[42] Leggett A, Zarit SH, Nguyen NH, Hoang CN, Nguyen HT. The influence of social factors and health on depressive symptoms and worry: A study of older Vietnamese adults. Aging Ment Health. 2012; 16: p. 780–6. doi: 10.1080/13607863.2012.667780 22486622 10.1080/13607863.2012.667780 PMC4233407

[43] Tran T V., Ngo D, Conway K. A cross-cultural measure of depressive symptoms among Vietnamese Americans. Soc Work Res. 2003; 27: p. 56–64.

[44] Sherbourne CD, Stewart AL. The MOS social support survey. Soc Sci Med. 1991; 32: 705–14. 2035047 10.1016/0277-9536(91)90150-b

[45] Lim T, Zelaya C, Latkin C, Quan VM, Frangakis C, Ha TV, et al Individual-level socioeconomic status and community-level inequality as determinants of stigma towards persons living with HIV who inject drugs in Thai Nguyen, Vietnam. J Int AIDS Soc. 2013; 16: p. 18637 doi: 10.7448/IAS.16.3.18637 24242257 10.7448/IAS.16.3.18637 PMC3833190

[46] Babor TF, Higgins-Biddle JC, Saunders JB, Monteiro MG. The Alcohol Use Disorders Identification Test: guidelines for use in primary care 2nd edition. World Health Organization; 2001.

[47] Li L, Tuan NA, Liang L-J, Lin C, Farmer SC, Flore M. Mental health and family relations among people who inject drugs and their family members in Vietnam. Int J Drug Policy. 2013; 24: p. 545–9. doi: 10.1016/j.drugpo.2013.06.007 23910167 10.1016/j.drugpo.2013.06.007 PMC3872211

[48] Vu NTT, Holt M, Phan HTT, La LT, Tran GM, Doan TT, et al Amphetamine-type-stimulants (ATS) use and homosexuality-related enacted stigma are associated with depression among men who have sex with men (MSM) in two major cities in Vietnam in 2014. Subst Use Misuse. 2017; 52: p. 1411–9. doi: 10.1080/10826084.2017.1284233 28436758 10.1080/10826084.2017.1284233

[49] Hershow RB, Zuscov DS, Nguyen VTM, Chander G, Hutton HE, Latkin C, et al “[Drinking is] like a rule that you can’t break”: A qualitative study on perceived barriers and facilitators to reduce alcohol use and improve antiretroviral treatment adherence among people living with HIV with alcohol use disorders in Thai Nguyen, Vietnam. Subst Use Misuse. 2018; Forthcoming. 10.1080/10826084.2017.1392986 PMC6198809 29537932

[50] Almog YJ, Anglin MD, Fisher DG. Alcohol and heroin use patterns of narcotics addicts: gender and ethnic differences. Am J Drug Alcohol Abuse. 1993; 19: p. 219–38. 8484358 10.3109/00952999309002682

[51] Anglin MD, Almog IJ, Fisher DG, Peters KR. Alcohol use by heroin addicts: evidence for an inverse relationship. A study of methadone maintenance and drug-free treatment samples. Am J Drug Alcohol Abuse. 1989; 15: p. 191–207. 2729226 10.3109/00952998909092720

